# Unmasking Cybercrime with Artificial-Intelligence-Driven Cybersecurity Analytics

**DOI:** 10.3390/s23146302

**Published:** 2023-07-11

**Authors:** Amir Djenna, Ezedin Barka, Achouak Benchikh, Karima Khadir

**Affiliations:** 1 College of New Technologies of Information and Communication, University of Constantine 2, Constantine 25000, Algeria; achouak.benchikh@univ-constantine2.dz (A.B.); karima.khadire@univ-constantine2.dz (K.K.); 2 College of Information Technology, United Arab Emirates University, Al Ain P.O. Box 17555, United Arab Emirates; ebarka@uaeu.ac.ae

**Keywords:** artificial intelligence, cyber threat intelligence, digital forensics investigation, cyber criminality, cybersecurity analytics

## Abstract

Cybercriminals are becoming increasingly intelligent and aggressive, making them more adept at covering their tracks, and the global epidemic of cybercrime necessitates significant efforts to enhance cybersecurity in a realistic way. The COVID-19 pandemic has accelerated the cybercrime threat landscape. Cybercrime has a significant impact on the gross domestic product (GDP) of every targeted country. It encompasses a broad spectrum of offenses committed online, including hacking; sensitive information theft; phishing; online fraud; modern malware distribution; cyberbullying; cyber espionage; and notably, cyberattacks orchestrated by botnets. This study provides a new collaborative deep learning approach based on unsupervised long short-term memory (LSTM) and supervised convolutional neural network (CNN) models for the early identification and detection of botnet attacks. The proposed work is evaluated using the CTU-13 and IoT-23 datasets. The experimental results demonstrate that the proposed method achieves superior performance, obtaining a very satisfactory success rate (over 98.7%) and a false positive rate of 0.04%. The study facilitates and improves the understanding of cyber threat intelligence, identifies emerging forms of botnet attacks, and enhances forensic investigation procedures.

## 1. Introduction

**Figure 1:**
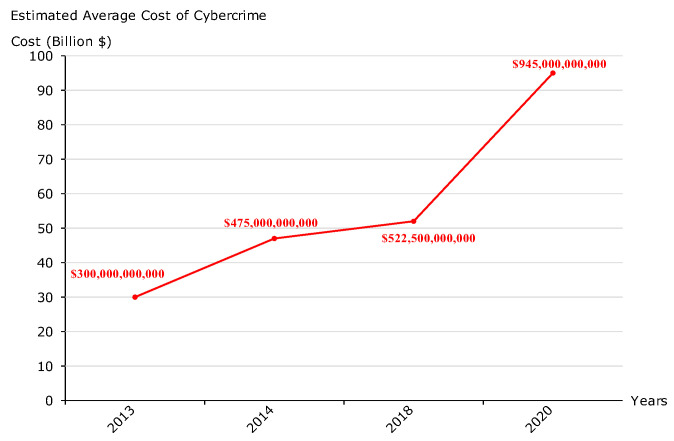
The average cost of cybercrime.

**Figure 2:**
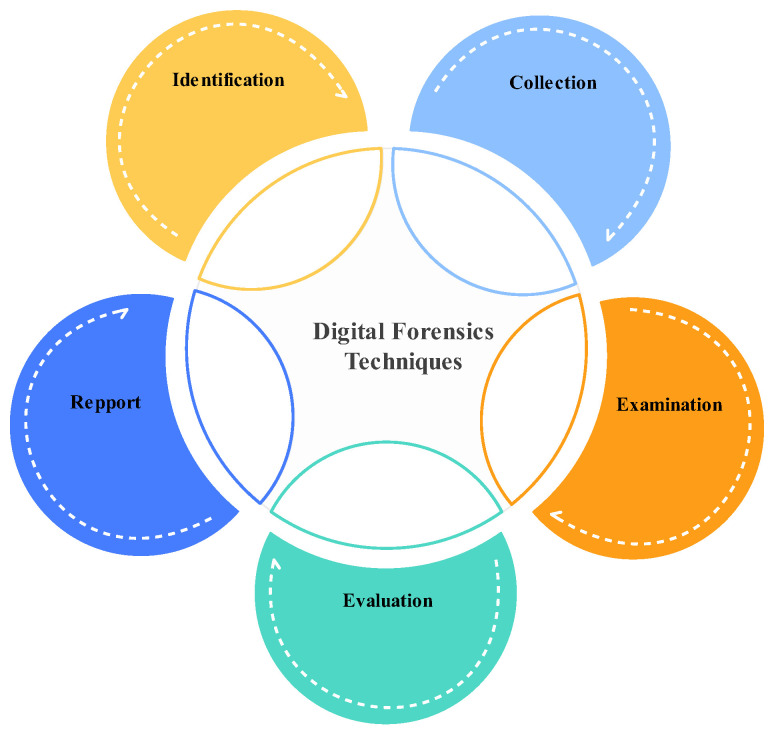
Procedure of digital forensics techniques.

Today’s top elite cyberattackers are competing in ingenuity to penetrate critical infrastructure, to conduct cyber espionage, and to exfiltrate sensitive data while covering their tracks. They use a variety of advanced and furtive techniques, scanning and exploiting numerous cyber vulnerabilities, creating hidden backdoors, and giving rise to several cyber threats. For instance, in 2017, NotPetya [1] affected several multinational companies by shutting down hundreds of thousands of machines in just ten minutes, resulting in some suffering losses of over USD 300 million. According to the Cyberwarfare Special Report [2], cybercrime is predicted to reach USD 10.5 trillion annually by 2025, which is exponentially higher than the damage inflicted by natural disasters in a year. According to Symantec [3], the top ten biggest cyber threats are phishing (22%), malware (20%), cyberattacks (to disrupt) (13%), cyberattacks for stealing money (12%), fraud (10%), cyberattacks for stealing IP (8%), spam (6%), internal attacks (5%), natural disasters (2%), and espionage (2%). According to the FBI [4], the Internet Crime Complaint Center reported that the volume of complaints in 2021 was 847,376, with losses of USD 6.9 billion. According to McAfee [5], up to 1% of the world’s GDP is now being lost to cybercrime. In addition, the cost of cybercrime to the global economy has increased by more than 50% in two years. Figure 1 presents the estimated average cost of cybercrime [6]:

Addressing cybercrime requires substantial research in cybersecurity analytics, cyber threat intelligence, and digital forensics. Digital forensics (DF) is a branch of forensic science that involves the recovery and investigation of digital devices and is often related to cybercrime. Digital forensic investigators use a variety of techniques to recover evidence from seized or damaged electronic devices in order to help identify criminals and to solve crimes [7]. Forensic analysis techniques can extract hidden and encrypted information using voiceprint analysis, darknet analysis, cryptanalysis techniques, and cybersecurity analytics. Cybersecurity analytics (CA) refers to the application of analytical techniques and technologies to analyze and interpret vast amounts of data in order to identify and respond to cybersecurity threats and risks. Cyber threat intelligence (CTI) refers to the gathered and analyzed information and knowledge about potential cyber threats and adversaries. It involves gathering intelligence, and evaluating and interpreting data from open-source intelligence (OSINT), the dark web, and incident reports so as to identify cyber threats and their capabilities, intentions, and potential targets. The technical aspects of these investigations are divided into several branches related to the types of digital devices involved: system forensics [8], database forensics [9], email forensics [10], malware forensics [11], memory forensics [10], file system forensics [8], mobile devices forensics [10], network forensics [8,10], digital image forensics [8], web forensics [8], cloud forensics [8], and IoT forensics [12]. Figure 2 illustrates the digital forensics investigation process.

For instance, in the field of IoT forensics, investigation mandates the inspection of all traffic forms that are suspected of demonstrating botnet behavior.

Given the successful application of deep learning methods in various real-world scenarios, the LSTM-CNN model is proposed for the early detection of botnet attacks. The key contributions of this paper are as follows:

1. A deep learning model based on LSTM-CNN is proposed for investigating botnet traffic detection, with a focus on indicators of compromise, to enhance digital forensics and cyber threat intelligence, thereby helping to provide effective responses to cybercrime.
2. By leveraging deep learning techniques, the proposed model has the potential to adapt to changing attack patterns and to learn intricate features automatically, thereby demonstrating adaptability to evolving advanced botnet techniques that evade detection.
3. This study aims to discover hidden patterns and correlations in botnet activities that may not be apparent using traditional approaches. This is crucial in enhancing cyber threat intelligence and in facilitating proactive forensic measures.

The remainder of this paper is organized as follows: Section 2 emphasizes the most common related work for botnet detection. Section 3 presents our proposed work, outlining our methodology for enhancing digital forensics techniques using DL models. The experimental results are described in Section 4, followed by the discussions. Finally, Section 5 provides a conclusion and perspectives.

## 2. Related Work

In the 21st century, the rise of advanced malware and the exploitation of zero-day vulnerabilities have become major cybersecurity concerns, affecting diverse sectors. The pervasive nature of these threats has drawn considerable attention from security experts, specialists, and researchers. As a result, numerous researchers are actively working towards finding effective solutions to combat these pressing issues.

In this research study, our focus lies in investigating IoT botnets, and several related studies are examined. A notable contribution is presented in Reference [13], which introduced a convolutional neural network (CNN)-based approach for the detection and classification of IoT botnets. Ge et al. recently put forth a study [14] employing deep learning (DL) techniques for intrusion detection in IoT networks. Their proposed model utilizes feed-forward neural networks (FNN) for the binary and multi-class classification of diverse attacks targeting IoT devices, employing the Bot-IoT dataset; however, while the suggested solution yielded satisfactory outcomes overall, the authors acknowledged the challenges encountered during the evaluation process and offered suggestions for potential enhancements. In Reference [15], the authors put forth a detection model that combines a bidirectional recurrent long-term memory neural network (BLSTM-RNN) with word-embedding (WE) techniques. Similarly, another study on botnet detection utilizing deep learning (DL) is presented in Reference [16]. In this research, an extensive set of 650 experiments was conducted on a substantial dataset of 83 GB, which was generated by merging existing datasets containing botnet and peer-to-peer (P2P) data traffic. The primary objective of this investigation was to assess the ability of DL to identify known botnets, including Zeus, Storm, Waledac, and ZeroAccess, and to potentially replace traditional detection methods reliant on network statistics and feature engineering.

Popoola et al. [17] presented a detection algorithm that relies on deep recurrent neural networks (DRNN). Their algorithm was evaluated using the Bot-IoT dataset, and the results were remarkable. Hegde et al. [18] directed their attention towards the identification of botnets through the implementation of multiple machine learning and deep learning classifiers. The IoT-23 dataset, exclusively comprising botnet data, along with benign data captured in a controlled test environment, served as their primary data sources, using a combination of four malware captures and three benign data captures solely from the IoT-23 dataset [19].

In the study conducted by Garcia et al. [20], the authors examined the labeling process employed in the CTU13 dataset. They emphasized that the labeling process guarantees the accurate classification of flows as normal or botnet. However, it was noted that flows labeled as Background may include traffic from both categories. Consequently, each CTU13 dataset contains an unlabeled segment that necessitates additional investigation [21]. Geetha K. and Brahmananda S.H. [22] directed their efforts towards developing a method to safeguard healthcare IoT devices against botnet attacks. The proposed algorithm incorporates bidirectional long short-term memory (BLSTM). This outcome demonstrated the capability of the algorithm in accurately predicting and mitigating botnet attacks within healthcare IoT networks, showcasing its potential in enhancing the security of such systems.

Table 1 is a summary encompassing recent research endeavors that primarily concentrate on IoT botnet forensics and detection:

Despite the presence of numerous botnet detection techniques, a research gap persists in the development of precise and efficient models capable of effectively addressing both the spatial and temporal aspects of botnet activities. Existing methods predominantly focus on the analysis of network traffic, leaving a dearth of research that explores the intricate dynamics of botnet behavior. This research gap highlights the need for comprehensive exploration of the effectiveness of AI models in mitigating the challenges posed by the dynamic botnet behavior and zero-day botnet attacks.

Moreover, in light of the escalating proliferation of insecure IoT devices, botnet attacks have emerged as a significant threat to internet security [33]. Numerous machine-learning-driven solutions have been put forth to detect diverse forms of botnet attacks. The effectiveness of these solutions predominantly relies on the selection of features employed to train the deep learning models. It is crucial to carefully consider the choice of features as they directly impact the performance and accuracy of the detection mechanisms. Nonetheless, the process of selecting features solely from a specific dataset imposes limitations on the capability of deep learning models to effectively detect botnet attacks across diverse datasets, given the variations in botnet attack samples. In this study, we propose a collection of universal features that can aid deep learning models in identifying various botnet attacks, irrespective of the dataset used. Further investigation is necessary to ascertain the optimal architectural design that can harness the strengths of both convolutional neural networks (CNN) and long short-term memory (LSTM) networks, with the aim of constructing a more robust and powerful botnet detection system.

## 3. Proposed Work

According to the Interpol Report 2021 [34], botnets are networks of compromised machines used to automate large-scale campaigns, including DDoS attacks, phishing, malware distribution, and data theft. Thus, detecting and mitigating botnet attacks is a critical task for ensuring the security of cyberspace. However, traditional-method-based botnet detection often relies on handcrafted features or rule-based approaches, which may struggle to keep up with the evolving techniques employed by botnet operators. Therefore, there is a need for advanced detection approaches that can effectively capture the complex patterns and dynamics of botnet attacks. This research gap necessitates investigating the effectiveness of the CNN-LSTM hybrid model in addressing the challenges associated with botnet detection. The application of the proposed CNN-LSTM hybrid model for botnet detection holds much research significance, such as enhanced detection accuracy, adaptability to evolving advanced botnet techniques, efficient feature learning and representation, comprehensive analysis of spatial and temporal contexts, real-time detection, and proactive forensics investigation measures.

Our approach was developed using the Google Colab and TensorFlow frameworks with the Python programming language. Python was chosen for its advantageous features, including concise coding requirements, extensive availability of libraries and frameworks, consistency, platform independence, a thriving community, and flexibility. These attributes collectively contribute to the efficiency and effectiveness of the implementation instead.

CNN (convolutional neural network) is widely acknowledged as one of the most successful deep learning methods. Its architecture comprises four key layers: the input layer, convolutional layer, pooling layer, and fully connected layer. CNNs can be structured as 1D CNN, 2D CNN, or 3D CNN, each catering to specific data types. For instance, 1D CNN is primarily utilized for processing sequence data, 2D CNN is commonly employed for image and text recognition tasks, while 3D CNN finds applications in medical image analysis and video data recognition. In this study, we adopt the 1D CNN variant [35], given its compatibility with the nature of our data.

**Figure 3:**
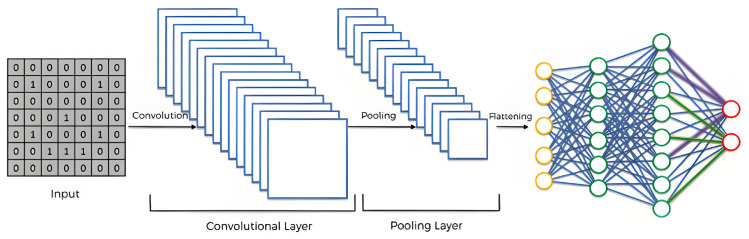
Structure of CNN model.

CNN (convolutional neural network) is a deep learning architecture commonly used for image and visual data analysis tasks. It is particularly effective in capturing spatial relationships and extracting meaningful features. This is an explanation of the operational principles behind a CNN model:

- Convolutional Layer: It consists of multiple learnable filters that slide across the input. Each filter performs a dot product operation between its weights and a small region of the input, producing a feature map. The feature map highlights important patterns or features present in the input.
- Activation Function: After the convolutional operation, an activation function is applied element-wise to the feature map. The activation function introduces non-linearity into the network, allowing it to learn complex relationships between the input and the extracted features.
- Pooling Layer: Following the activation function, a pooling layer is often applied. Pooling reduces the spatial dimensions of the feature maps while retaining important information. Pooling helps to reduce the number of parameters, to decrease computational complexity, and to provide translational invariance.
- Convolution and Pooling Layers: The convolutional and pooling layers are typically repeated multiple times in a CNN architecture to capture increasingly complex and abstract features. This allows the network to learn hierarchical representations of the input data, starting from simple low-level features and progressing to high-level features.
- Flattening: After the convolutional and pooling layers have been applied, the resulting feature maps are flattened into a one-dimensional vector. This flattening operation reshapes the multi-dimensional feature maps into a single continuous vector, which serves as the input to the subsequent fully connected layers.
- Fully Connected Layers: After flattening, fully connected layers are added to the network. These layers are similar to those found in traditional neural networks, where each neuron is connected to every neuron in the previous layer. Fully connected layers perform non-linear transformations on the input data and are responsible for making predictions based on the extracted features.
- Output Layer: The final layer of the CNN is the output layer. It typically consists of one or more neurons, depending on the specific task. Figure 3 [36] shows the schematic structure of the CNN model.

**Figure 4:**
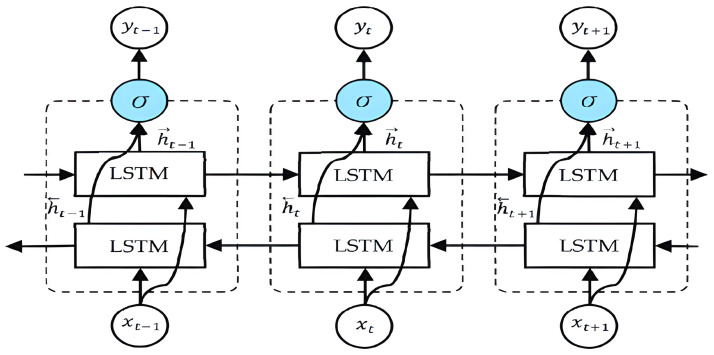
Structure of LSTM model.

LSTM (long short-term memory) networks are a specialized variant of recurrent neural networks that incorporate memory cells within hidden layers, enabling selective long-term pattern retention. This characteristic makes LSTM networks well-suited for modelling sequential data, which was the underlying motivation for our selection. Moreover, LSTM networks are employed to capture the intricate dynamics inherent in human activity, further highlighting their utility in analyzing complex temporal patterns [37]. It overcomes the limitations of traditional RNNs by addressing the vanishing gradient problem, which hampers the ability of the network to capture long-term dependencies in the data. The key components of an LSTM model are memory cells, gates, and input/output connections. This is an explanation of the operational principles behind an LSTM model:

- Input and Output: At each time step in the sequence, the LSTM receives an input vector. The input can be a single value or a vector of multiple values. The LSTM processes the input and produces an output vector at the same time step.
- Memory Cell: The memory cell is the core component of the LSTM. It maintains and updates its internal state based on the current input, the previous state, and the output of the previous time step. The memory cell has the ability to store and carry information over long durations, allowing the model to capture dependencies over time.
- Forget Gate: The forget gate determines which information from the previous state should be forgotten or discarded. It takes the previous output and current input as inputs, and using a sigmoid activation function, it produces a forget gate vector. This vector selectively removes or keeps information from the previous state.
- Input Gate: The input gate determines which new information should be stored in the memory cell. It takes the previous output and current input as inputs and produces an input gate vector. Additionally, it generates a candidate vector, which represents potential new information.
- Output Gate: The output gate decides what information from the memory cell should be outputted. It takes the previous output and current input as inputs and produces an output gate vector using a sigmoid activation function. The memory cell state is passed through a tanh activation function to squash the values, and then, the output gate vector is applied to filter the values.
- Output: The output is generated by combining the filtered memory cell state with the output gate vector. This output can be used for prediction or fed as an input to the next time step in the sequence. Figure 4 [38] shows the schematic structure of the LSTM model.

Our model proposes the CNN-LSTM algorithm, which combines the benefits of convolutional neural networks (CNNs) and long-term memory networks (LSTMs). The CNN-LSTM algorithm utilizes a shallow CNN to extract basic features from the dataset. The feature tensor extracted from the CNN is then converted into a feature matrix. Finally, the rows of the feature matrix are fed into the LSTM network to combine them, facilitating the implicit mapping of the entire dataset to the desired target.

Based on the evaluation of the CTU-13 and IoT-23 datasets, it is evident that the inclusion of CNN significantly enhances the feasibility of the CNN-LSTM algorithm. However, when it comes to the feature hybrid phase, LSTM emerges as the more effective component. The test results strongly indicate the valuable role played by CNN in enhancing the overall effectiveness of the algorithm, while highlighting the exceptional performance of LSTM in the specific context of the feature hybrid phase. Additionally, the botnet detection accuracy in IoT exceeds 90%.

Cyber investigators have been employing classification techniques grounded in artificial intelligence (AI), which are well-suited for effectively handling large volumes of data and processing them rapidly. Drawing inspiration from this approach, our aim is to propose a model founded on advanced AI techniques, specifically deep learning, to identify botnets as a significant source of cyberattacks. The optimal utilization of 1D CNN for both numeric and textual data, coupled with LSTM known for its memory capabilities, forms the basis of our model. We applied this model to two datasets, namely CTU13 and IoT23, which encompass diverse scenarios. To ensure compatibility with the model’s input requirements, we converted these datasets accordingly. Moreover, data balancing techniques such as SMOTE were employed to augment the volume of data and to mitigate any potential class imbalance. SMOTE (synthetic minority over-sampling technique) is a data balancing technique widely employed in machine learning to tackle class imbalance within datasets. This technique aims to address the scarcity of data in the minority class by oversampling it. It operates by identifying the nearest neighbors of each minority data point and by generating synthetic data points along the line segments that connect the minority data point to its nearest neighbors. Extensive research has demonstrated the effectiveness of SMOTE in enhancing the performance of machine learning models when dealing with imbalanced datasets.

### 3.1. CNN-LSTM Hybrid Model

In this section, we introduce the hybrid CNN-LSTM model that has been developed by combining CNN and LSTM to enhance the investigation process. We provide a comprehensive explanation of how this model is constructed, including its inputs, outputs, and the results obtained from our experiments.

#### 3.1.1. Data Source

A comprehensive framework for developing an IoT botnet detection model has been established. The framework encompasses the entire process, starting from defining the botnet datasets to the detection phase. This section focuses on the CTU-13 and IoT-23 datasets utilized within the framework and provides further details regarding the proposed framework.

##### CTU-13 Dataset

CTU-13 [39], also known as the Czech Technical University 13 dataset, is a dataset of botnet traffic commonly used for cybersecurity research and analysis. It was created by the Cyber Threat Intelligence Lab at the Czech Technical University in Prague. The purpose of the dataset was to provide a large collection of real botnet traffic mixed with normal and background traffic. The CTU-13 dataset consists of thirteen captures, each of which represents a different botnet sample. In each scenario, we ran specific malware that used multiple protocols and performed different actions. The CTU-13 dataset was designed to provide researchers and practitioners with a realistic and diverse collection of network traffic data for studying various aspects of cybersecurity. It focuses on capturing different types of network traffic, including normal traffic as well as traffic generated by various malware and botnet activities. The dataset was collected in a controlled environment using a network of virtual machines. It includes both benign traffic and traffic generated by 13 different malware families, covering a wide range of malicious activities. Including well-known ones such as Zeus, SpyEye, and Conficker, as well as lesser-known families. Each malware family represents a different attack scenario, allowing researchers to analyze and develop resilience investigation methods for specific types of malware. The table below shows the characteristics of the botnet scenarios. Each scenario was captured in a PCAP file that contains all packets of all three traffic types.

The PCAP files underwent processing to extract additional information, including NetFlows and WebLogs. The extracted information was then converted into CSV format using Wireshark. The resulting dataset possesses a multivariate and sequential data structure, comprising numerous instances and approximately 15 attributes. Given its size, not all of the data was utilized for model training and testing. Instead, specific instances were selected from the data folder to streamline model preparation.

However, using all instances could result in additional time consumption due to the substantial volume of data that needs to be processed. The data attributes, along with their corresponding details, are outlined below:

- StartTime: the start time for capturing data traffic;
- Dur: the duration of capture of data traffic or duration of the attack on the devices;
- Proto: the protocol used in the traffic;
- SrcAddr: the source IP address;
- Sport: the source port address;
- Dir: the direction of data flow and attack;
- DstAddr: the destination IP address;
- Dport: the destination port address;
- State: the state during the capture;
- dTos: the destination type of service;
- TotPkts: the total number of packets transferred or received during the capture;
- TotBytes: the total size of packets transferred or received during the capture in bytes;
- SrcBytes: size of packets from the source;
- Label: attack tag (indicating whether it was a successful, background, or normal botnet attack).

Table 2 illustrates the characteristics of the botnet scenarios.

##### IoT-23 Dataset

IoT-23 [40] is a publicly available dataset commonly used for research and analysis in the field of Internet of Things (IoT) security. It was created by the Stratosphere Laboratory at the Czech Technical University in Prague. The IoT-23 dataset was developed to provide researchers and practitioners with a comprehensive dataset for studying IoT security, particularly in the context of botnet detection. It focuses on capturing network traffic data generated by IoT devices and includes various botnet activities for analysis. The IoT-23 dataset includes instances of various botnet activities for each device type. These botnet activities are injected into the network traffic data, representing different types of botnet infections, command and control (C&C) communication, and malicious behavior exhibited by compromised IoT devices. This allows researchers to study and develop detection methods for IoT botnets. IoT-23 dataset’s focus on IoT devices reflects the growing security concerns surrounding IoT deployments. By studying botnet detection on IoT devices using this dataset, researchers can address the emerging security challenges and contribute to enhancing the security and resilience of IoT ecosystems. The dataset’s relevance to real-world IoT scenarios allows for practical and applicable findings. It consists of 23 captures of malware running in IoT devices and three captures of benign IoT device traffic, thus providing a large dataset of real and tagged IoT malware infections and benign IoT traffic for researchers to develop machine learning algorithms. The research on this dataset was funded by Avast. The malware was allowed to connect to the Internet.

The IoT-23 dataset is multi-tagged, and the tags belong to similar classes. The labels represent different attack types, but the classes can be a combination of different attacks. For instance, a label can be either C&C or PartOfAHorizontalPortScan, each having a different meaning. In contrast, a class can be C&C-PartOfAHorizontalPortScan, which indicates that both malware attacks are present for streams in that class. The specific details of the data attributes are presented below:

- Attack: The infected device attempts to take advantage of a vulnerability in another host as an attack.
- Benign: The connections do not show any suspicious or malicious activity.
- C&C: The infected device is connected to a Command & Control server.
- DDoS: The infected device executes a distributed denial of service (DDoS) attack.
- FileDownload: The infected device downloads a file.
- HeartBeat: The packets sent over this connection are used by the Command & Control server to keep track of the infected host.
- Mirai: The connections exhibit characteristics of a Mirai botnet.
- Okiru: The connections exhibit the characteristics of an Okiru botnet.
- PartOfAHorizontalPortScan: The connections are used to perform a horizontal port scan to gather information for potential future attacks.
- Torii: The connections have the characteristics of a Torii botnet.

The following tables, namely Table 3 and Table 4, provide a comprehensive overview of each scenario present in the IoT-23 dataset, along with their fundamental characteristics:

#### 3.1.2. Data Preparation

In this section, a comprehensive description of the dataset is presented, encompassing various aspects such as label matching, dataset extraction, feature extraction, pre-processing, experiment setup, and results analysis.

CTU-13 Dataset

The converted dataset has been uploaded in a CSV file format named ‘flowdata’, which has been normalized and standardized based on the CTU 13 dataset.

IoT-23 Dataset

To preprocess the IoT-23 dataset, the process involves loading 16 individual datasets from the original 23 datasets into a Pandas database. The first 10 rows (headers) are skipped, and the subsequent 100,000 rows are loaded. Once this process is completed for all 16 datasets, they are combined to create a new dataset called “IoT23-combined.csv”.

Once the data are loaded using the pandas library’s “read-csv()” function, labeling becomes necessary. To accomplish this, a function named “labeler()” is employed. The function iterates through each line of the data, checking the “Label” column, and replacing the string values with the corresponding labels of 0 and 1, or possibly 0.1 and 2.

The pre-processing phase involves several crucial steps, including the elimination of noisy and redundant data, as well as the normalization and transformation of the dataset. These pre-processing procedures are executed on both the training and testing data, ensuring consistency and reliability throughout the entire data analysis process. By removing noise and unused data and by applying appropriate normalization and transformation techniques, the dataset is prepared to enhance the accuracy and effectiveness of subsequent data analysis and modeling tasks.

#### 3.1.3. Model Architecture

**Figure 5:**
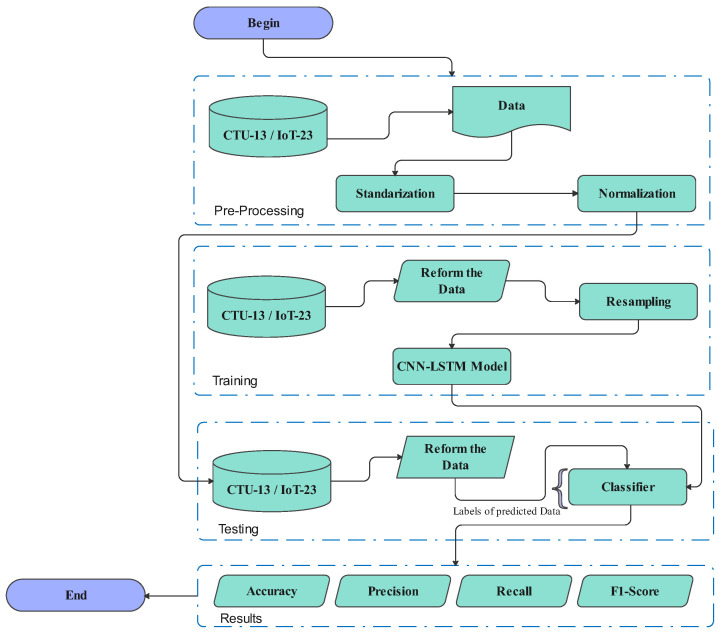
Architecture of the proposed system.

The following Figure 5 illustrates the architecture of our approach.

The Algorithm 1 below shows the CNN-LSTM model we used:
**Algorithm 1** Pseudo-code of CNN-LSTM1:Input the trained data as a CSV file from datasets.2:Scaling and transforming all the data features for learning.3:Scaling and transforming all features of the testing data.4:Using SMOTE for data balancing.5:Definition of the CNN-LSTM model:6:| The body of the model (layers)7:Return model8: $Model\leftarrow CNN_{-}LSTM\left(\right)Model$9:Fitting function10:Valuation calculation

SMOTE (synthetic minority over-sampling technique) is a data augmentation technique that commonly uses deep learning to address the problem of class imbalance. Class imbalance occurs when the number of instances in one class is significantly lower than the number of instances in another class, leading to biased learning and reduced performance. The SMOTE technique aims to alleviate class imbalance by synthesizing new instances for the minority class. Class imbalance can lead to biased models that tend to favor the majority class and to perform poorly on the minority class. This is achieved by identifying the nearest neighbors of each minority class instance and by creating synthetic instances along the line segments connecting them. This is carried out by taking the difference between feature values of the instance and its neighbor, multiplying it by a random number between 0 and 1 and adding the result to the instance’s feature values. Therefore, by applying SMOTE to the botnet detection dataset, we can alleviate the class imbalance issue and can enhance the model’s ability to detect botnet traffic accurately. The generated synthetic instances provide additional training examples for the minority class, enabling the deep learning model to learn from a more balanced dataset and to improve its overall performance.

#### 3.1.4. Model Structures

**Figure 6:**
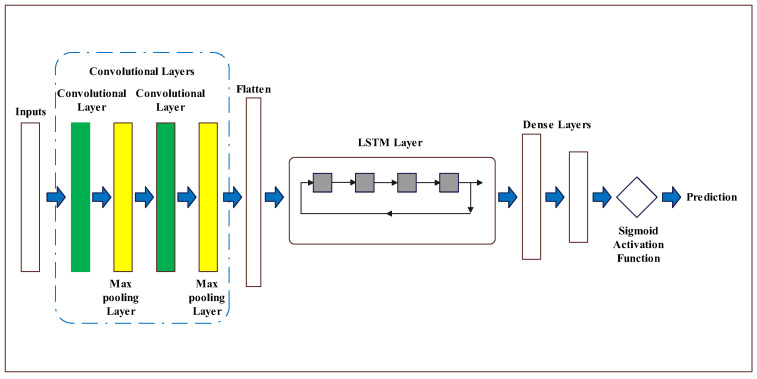
The structure of the layers used.

Our approach consists of two CNN layers, which are separated by Dropout and followed by an LSTM layer. The configuration and sequence of these layers are visually depicted in Figure 6 to provide a clearer understanding.

**Figure 7:**
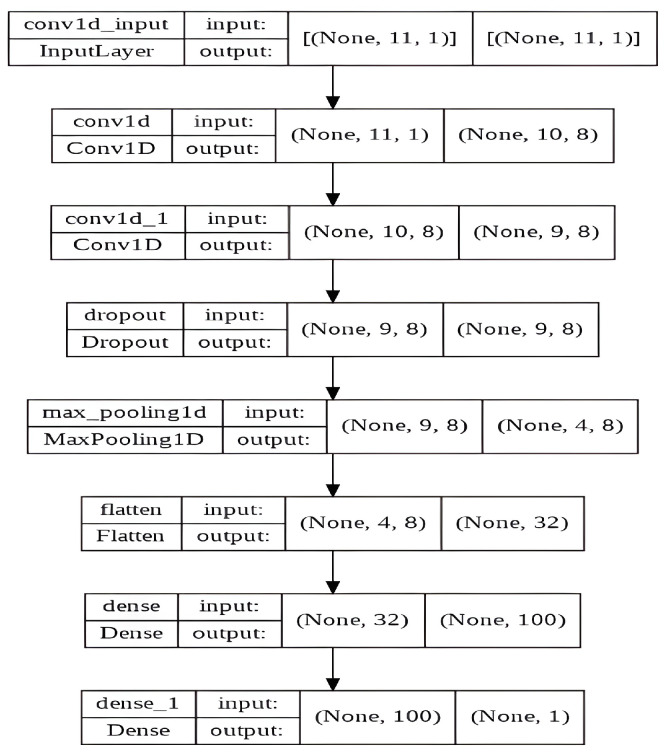
Internal structure of the proposed CNN model.

To implement the aforementioned architecture, we utilized the internal structure of the CNN model illustrated in Figure 7.

The data were partitioned, allocating 80% for training and 20% for testing. Consistent methodologies were applied for both training and testing across the two datasets originating from distinct sources. Given that each model exclusively utilizes either the IoT-23 or CTU-13 dataset, the stratification method was employed to ensure the preservation of the original distribution of training values, thereby safeguarding against any potential alterations. A simplified representation of this method is outlined below:

Algorithm 2 below shows the global steps of preprocessing, model architecture, model training, model evaluation, model testing, and model deployment we used:
**Algorithm 2** Global steps of preprocessing, training, testing, and deployment1:Collect and preprocess the data on botnet related malware samples.2:Convert the raw data into suitable format for model input.3:Define the hybrid CNN-LSTM model. (Input layer: Receive preprocessed data, CNN layer: Extract spatial features from dataset, LSTM layer: Capture temporal dependencies in the data sequences, Output layer: Perform prediction of botnet or non-botnet classes).4:Split the data into training, validation, and test sets.5:Initialize the model’s neuron weights.6:Train the model.7:Pass the training data through the model.8:Adjust the model weights using gradient backpropagation to minimize prediction error.9:Repeat these steps on the training data until maximum performance is achieved.10:Evaluate the model.11:Use the validation data to assess the model’s performance on unseen data.12:Measure performance metrics (accuracy, recall, F1-score, etc.).13:Test the model.14:Use the test data to evaluate the finale performance of the model.15:Analyse the results to assess the effectiveness of botnet detection.16:Utilize the trained model.17:Apply the model in real-time to detect suspicious botnet activities in new traffic data.18:Integrate the model into existing botnet detection and security tools to enhance forensics investigation capabilities.

## 4. Experimental Results

In this section, we assess the effectiveness of the CNN and LSTM models in terms of their robustness against underfittingand overfitting, as well as their generalization ability. This evaluation aims to gauge the model performance and to ascertain their suitability for broader applications beyond the training data. The confusion matrix can have four different elements. Some of the commonly used performance metrics [41] for data classification are discussed below.

True Positive (TP): The classifier accurately identified the attack’s class characteristics. It implies that a classifier, which is a type of machine learning algorithm, was used to identify the type of attack that was detected, and that it did so correctly.

True Negative (TN): The value of the class characteristic is negative, i.e., normal traffic.

False Positive (FP): The classifier wrongly classifies normal traffic as an attack.

Faux Negative (FN): The classifier misclassifies an attack record as normal traffic.

### 4.1. Accuracy (Success Rate)

Accuracy, also known as the success rate, indicates the percentage of normal activities and attacks that are correctly detected. It is calculated using the ratio between the correct detection and the total detection. It is calculated as follows:

(1) Accuracy=TP+TN/TP+TN+FP+FN

### 4.2. Precision

This metric shows the percentage of detected attacks that are actually real attacks. It is calculated as follows:

(2) Precision=TP/TP+FP

### 4.3. False-Positive Rate (FPR)

This indicates the percentage of false alarms, which is obtained by calculating the ratio between the number of traffic incorrectly classified as intrusions and the total number of normal traffic. It is calculated as follows:

(3) FPR=FP/TN+FP

### 4.4. Recall (Detection Rate)

This indicates the percentage of attacks detected compared with all attacks presented in the dataset. It is the ratio between the number of correctly detected intrusions and the total number of intrusions, i.e., how many positives the model identified among all possible positives. It is calculated as follows:

(4) Recall=TP/TP+FN

### 4.5. F-Score (Harmonic Mean)

The harmonic mean F combines recall and precision into a number between 0 and 1. It is calculated as the mean of precision and recall, given by

(5) F-Score=2×(Precision×Recall)/(Precision+Recall)

Table 5 illustrates the training of the CNN model, where the highest accuracy obtained is 99.7%, and the best fault tolerance result is 0.04%. This was achieved by adding a convolutional layer and a dropout layer with a dropout rate of 0.5.

Table 6 displays the training results of the hybrid model. As depicted, an accuracy of 98.74% and a fault tolerance of 0.04% were achieved.

**Figure 8:**
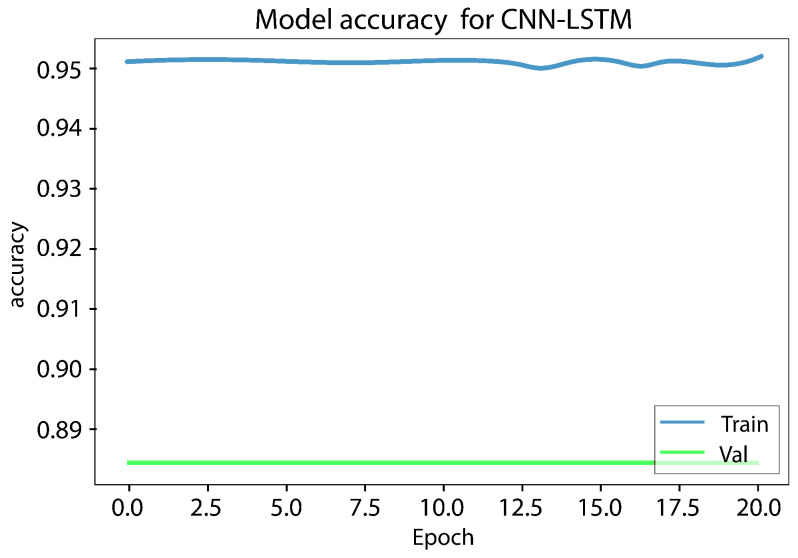
Accuracy underfitting result of the CNN LSTM model of the IoT 23 dataset.

**Figure 9:**
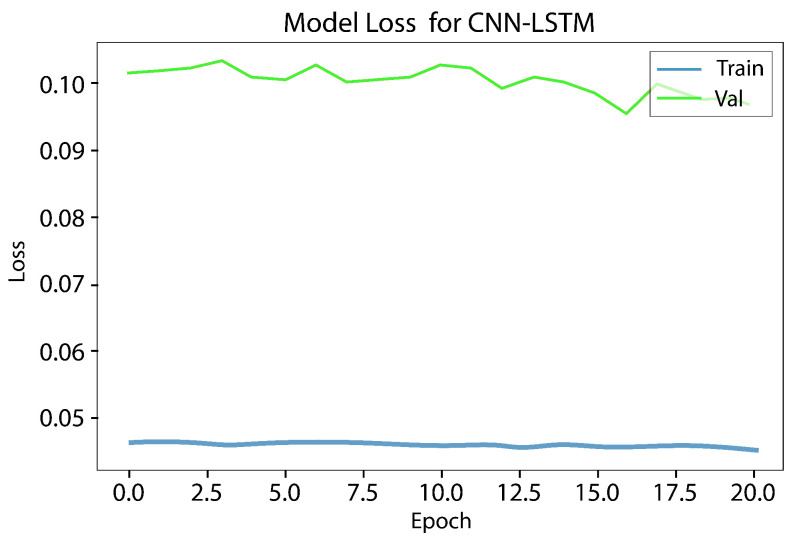
Loss underfitting result of the CNN LSTM model of the IoT 23 dataset.

**Figure 10:**
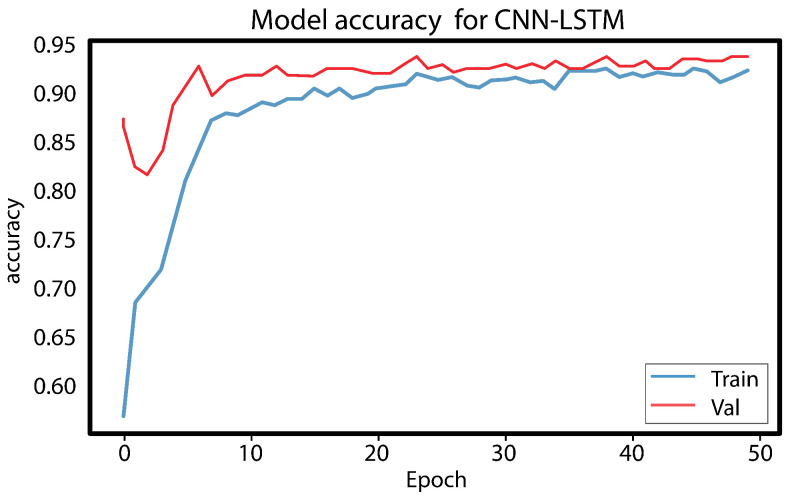
Accuracy training and validation.

**Figure 11:**
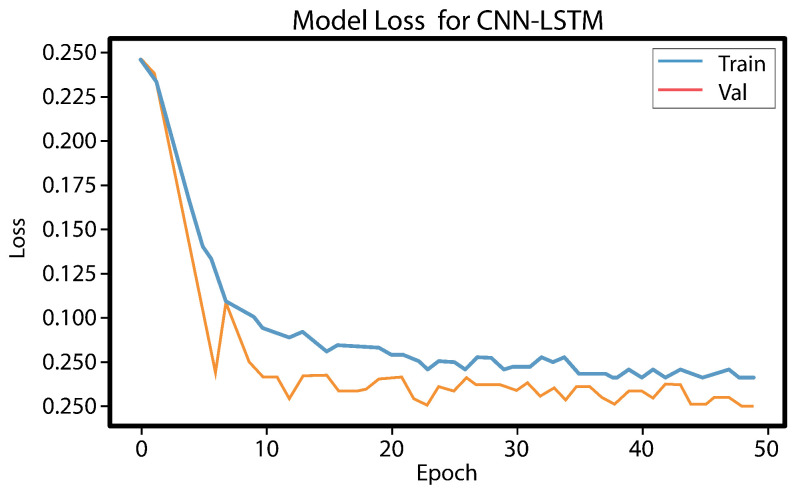
Loss training and validation.

The LSTM model underwent testing, and it took up to 10 h to obtain a result for a single function. However, since a result for seven or more functions was needed, it was not a practical choice. Digital forensics investigators need to collect and disclose evidence quickly, and therefore, time is the most important element. Hence, LSTM was excluded from the conducted comparison. The experiment results show that using the best features reduces the training time and provides a high rate of bot detection. Figure 8 and Figure 9, illustrates the results obtained from the CNN LSTM model applied to the IoT 23 dataset, which indicate the presence of loss underfitting. Further analysis and adjustments to the model training process are required to address the underfitting issue, such as increasing the model’s complexity, augmenting the training data, applying regularization techniques, fine-tuning the hyperparameters, utilizing model ensembles, incorporating pretrained models, expanding the training data size, and implementing early stopping and model checkpointing. Figure 10 and Figure 11 demonstrate that our approach achieves higher precision and competitive accuracy.

Initially, a combination of a CNN layer and an LSTM layer, followed by a dropout layer, was employed, but the obtained results were unsatisfactory. To enhance the performance, an additional CNN layer was incorporated, which yielded satisfactory outcomes. However, concerns regarding overfitting and underfitting were observed, as evident from the curves in Figure 8 and Figure 9. To mitigate these issues, another dropout layer was introduced, leading to noticeable changes in the observed patterns, as depicted in Figure 10 and Figure 11.

Figure 10 and Figure 11 show that the model performs well based on the success rate and validation loss. The success rate curve is lower than the validation curve, while the loss curve is higher than the validation loss curve.

Table 7 shows the system performance comparison of the proposed method and other works.

The metrics shown in Table 7 indicate that our proposed solution outperforms most of the state-of-the-art works. This method yields a much higher accuracy and fault detection rates than most of the previous works did on the CTU-13, IoT-23, and other datasets.

By using convolutional layers, pooling layers, fully connected layers, and backpropagation, CNNs can effectively learn and extract relevant features and their hierarchical structure, and can capture local patterns. Furthermore, by combining CNN and LSTM, the hybrid model can effectively learn and capture long-term dependencies in sequential data. The gates allow the model to selectively retain or discard malicious information, to update the memory cell state, and to generate appropriate outputs. This makes the CNN-LSTM model a well-suited and powerful tool for specific tasks such as malicious traffic classification, modern malware recognition, cyber threat intelligence, and other investigations involving cybersecurity analytics. This study also facilitates forensics analysis to enhance our understanding of cyber threat intelligence, to identify emerging forms of botnet attacks, and to enhance digital investigation procedures.

## 5. Conclusions

This research attempts to better understand the motivations driving cybercriminals, the techniques they employ, and the modus operandi behind botnet attacks. The proposed CNN-LSTM model can capture both spatial and temporal patterns in botnet activities, leading to improved accuracy in identifying and distinguishing normal network traffic from complex malicious botnet behavior. This can help reduce false positives and increase the precision of botnet detection systems. The model is constructed by integrating the CNN and LSTM layers, utilizing diverse information extracted from multiple scenarios found in the CTU-13 and IoT-23 datasets. Moreover, the proposed approach has the potential to operate in real time, enabling timely detection and response to botnet attacks with flexibility, and the capability to tackle complex and evolving cyber threats. This is crucial in enhancing cyber threat intelligence and facilitating proactive forensics measures. The results revealed that adopting the hybrid CNN-LSTM model led to a significant improvement in accuracy, yielding highly satisfactory performance outcomes. Our approach achieved an accuracy rate, exceeding 98% with a false-positive rate of 0.04%. Consequently, this study enables the model to effectively analyze complex cyber threats that exhibit both spatial and temporal characteristics, thereby enhancing cyber threat intelligence and facilitating proactive forensic measures.

In future works, our objective is to explore techniques to improve the robustness of deep learning models against adversarial attacks in the context of botnet detection. Adversarial attacks can be used by botnets to evade detection systems, so developing defenses against adversarial attacks is crucial. This endeavor aims to establish a robust framework capable of addressing a broader range of cyber threats. Furthermore, we attempt to explore the use of blockchain to facilitate secure and decentralized sharing of threat intelligence among different entities. Blockchain can enable the creation of a trusted and immutable ledger that allows organizations to share information about botnets in real time, improving the overall detection and mitigation efforts.

## Figures and Tables

**Table 1:** IoT botnet forensics- and detection-related work.

Work	Year	Journal	Method	Pros	Cons
[23]	2020	High Speed Networks	ML	Effective in detecting patterns and anomalies	May require a large labled dataset
[24]	2020	Applied Sciences	ML, DL	Capable of learning complex patterns	High computational complexity
[25]	2020	Security and Communication Networks	ML	Can identify hidden patterns and correlations	Limitations in handlings new attacks
[26]	2021	IEEE Internet of Things	Federated DL	Detection of zero-day botnet attacks	Synchronization and communication challenges
[27]	2021	SN Computer Science	DL	Can learn intricate features	Requires large amounts of labled data
[28]	2022	Ambient Intelligence and Humanized Computing	Game theory, DL	Models the strategic behavior of attackers	Requires extensive computational resources
[29]	2022	IEEE INFOCOM	Extreme learning	Fast and efficient learning	Requires fine-tuning for optimal performance
[30]	2023	Computers & Security	BiGRU-RNN	Improved accuracy in detecting IoT botnet attacks	Has increased complexity and resources requirements
[31]	2023	Computer Science	SVM	Adaptable to dynamic botnet	Requires extensive computation resources
[32]	2023	Future Generation Computer Systems	Active learning	Minimizes the labeling cost for the IoT botnet detection	Did not explore the implications and relation of specific features

**Table 2:** Characteristics of botnet scenarios.

Id	IRC	SPAM	CF	PS	DDoS	FF	P2P	US	HTTP
1	X	X	X						
2	X	X	X						
3	X			X				X	
4	X				X			X	
5		X		X					X
6				X					
7									X
8				X					
9	X	X	X	X					
10	X				X			X	
11	X				X			X	
12							X		
13		X		X					X

**Table 3:** Scenarios in IoT-23 dataset.

Scenarios	Type	Capture Name	Malware/Device	Duration	Number of Packets	Total Flows
Scenario 1	Malicious	CTU-IoT-Malware-Cap-34-1	Mirai	24,000	233,000	23,146,000
Scenario 2	Malicious	CTU-IoT-Malware-Cap-43-1	Mirai	1000	82,000,000	67,321,810,000
Scenario 3	Malicious	CTU-IoT-Malware-Cap-44-1	Mirai	2000	1,309,000	238,000
Scenario 4	Malicious	CTU-IoT-Malware-Cap-49-1	Mirai	8000	18,000,000	5,410,562,000
Scenario 5	Malicious	CTU-IoT-Malware-Cap-52-1	Mirai	24,000	64,000,000	19,781,379,000
Scenario 6	Malicious	CTU-IoT-Malware-Cap-20-1	Torii	24,000	50,000	3,210,000
Scenario 7	Malicious	CTU-IoT-Malware-Cap-21-1	Torii	24,000	50,000	3,287,000
Scenario 8	Malicious	CTU-IoT-Malware-Cap-42-1	Trojan	8000	24,000	4,427,000
Scenario 9	Malicious	CTU-IoT-Malware-Cap-60-1	Gagfyt	24,000	271,000,000	3,581,029,000
Scenario 10	Malicious	CTU-IoT-Malware-Cap-17-1	Kenjiro	24,000	109,000,000	54,659,864,000
Scenario 11	Malicious	CTU-IoT-Malware-Cap-36-1	Okiru	24,000	13,000,000	13,645,107,000
Scenario 12	Malicious	CTU-IoT-Malware-Cap-33-1	Kenjiro	24,000	54,000,000	54,454,592,000

**Table 4:** Scenarios in IoT-23 dataset.

Scenarios	Type	Capture Name	Malware/Device	Duration	Number of Packets	Total Flows
Scenario 13	Malicious	CTU-IoT-Malware-Cap-8-1	Hakai	24,000	23,000	10,404,000
Scenario 14	Malicious	CTU-IoT-Malware-Cap-35-1	Mirai	24,000	46,000,000	10,447,796,000
Scenario 15	Malicious	CTU-IoT-Malware-Cap-48-1	Mirai	24,000	13,000,000	3,394,347,000
Scenario 16	Malicious	CTU-IoT-Malware-Cap-39-1	IRCBot	7000	73,000,000	73,568,982,000
Scenario 17	Malicious	CTU-IoT-Malware-Cap-7-1	Linux, Mirai	24,000	11,000,000	11,454,723,000
Scenario 18	Malicious	CTU-IoT-Malware-Cap-9-1	Linux, Hajime	24,000	6,437,000	6,378,294,000
Scenario 19	Malicious	CTU-IoT-Malware-Cap-3-1	Muhstik	36,000	496,000	156,104,000
Scenario 20	Malicious	CTU-IoT-Malware-Cap-1-1	Hide and Seek	112,000	1,686,000	1,008,749,000
Scenario 21	Benign	CTU-Honeypot-Cap-7-1	Soomfy Doorlock	1400	8276	139,000
Scenario 22	Benign	CTU-Honeypot-Cap-4-1	Phillips HUE	24,000	21,000,000	461,000
Scenario 23	Benign	CTU-Honeypot-Cap-5-1	Amazon Echo	5400	398,000,000	1,383,000

**Table 5:** CNN model training results.

	Dataset	Without Sampling	CallBacks	Random under Sampler	SMOTE	SMOTE Tomek	Borderline SMOTE	ADASYN
Accuracy	CTU13	0.997520	0.998044	0.971556	0.997805	0.995140	0.993538	0.993538
	IoT23	0.952365	0.945287	0.892822	0.896551	0.945287	0.892836	0.896751
Precision	CTU13	0.886515	0.868871	0.195595	0.761460	0.588351	0.701167	0.517816
	IoT23	0.736959	0.845727	0.995560	0.995560	0.997780	0.999970	0.999989
Recall	CTU13	0.736959	0.845727	0.995560	0.995560	0.997780	1	1
	IoT23	0.991621	1	0.886644	0.890587	1	0.886660	0.890785
F-Score	CTU13	0.804848	0.857143	0.326955	0.862915	0.740222	0.824337	0.682317
	IoT23	0.975221	0.971874	0.939904	0.942116	0.971874	0.939912	0.942233
FPR	CTU13	0.000659	0.000892	0.028612	0.002179	0.004879	0.002978	0.006507
	IoT23	0.725878	1	0.000437	0.000402	1	0.000455	0.000175

**Table 6:** CNN-LSTM model training results.

	Dataset	Without Sampling	CallBacks	Random under Sampler	SMOTE	SMOTE Tomek	Borderline SMOTE	ADASYN
Accuracy	CTU13	0.997135	0.991959	0.935047	0.987422	0.977201	0.972064	0.972064
	IoT23	0.944316	0.889367	0.945266	0.892805	0.962097	0.878699	0.892822
Precision	CTU13	0.872011	0.455946	0.091452	0.330241	0.224216	0.190368	0.195288
	IoT23	0.958250	0.995851	0.945288	0.999970	0.154402	0.995798	0.958250
Recall	CTU13	0.688124	0.821310	0.935627	0.790233	0.928968	0.970936	0.930078
	IoT23	0.983963	0.886658	0.999976	0.886627	0.996670	0.875372	0.983963
F-Score	CTU13	0.769231	0.586371	0.166617	0.465816	0.361243	0.970936	0.316048
	IoT23	0.970936	0.938087	0.971863	0.939894	0.267381	0.931710	0.970936
FPR	CTU13	0.000706	0.006849	0.064957	0.011200	0.022462	0.000892	0.027643
	IoT23	0.740679	0.063824	0.999965	0.000455	0.038144	0.063824	0.970936

**Table 7:** Comparative study.

Work	Year	Method	Dataset	Accuracy
[42]	2018	PSI Graph CNN Classifier	IoTPOT-IotBotnet	92%
[43]	2019	Decision Tree	CTU-13	97.54%
[44]	2019	MEFC	Real life dataset	87.04%
[45]	2019	Hybrid feature selection	NSL-KDD UNSW-NB15	91.27%
[23]	2020	Reinforcement learning	ISOT, P2P, ISCX	98.3%
[46]	2021	Representativeness-based instance selection	KDD Cup 99	94.25%
[47]	2021	Sparse autoencoder	NSL-KDD CIC-IDS2017 AWID	98.10%
[29]	2022	Extreme learning	MedBIoT	97.7%
[30]	2023	SVM DT MLP	CTU-13	92%
[32]	2023	Active learning	MedBIoT	97%
[48]	2023	BiGRU-RNN	IoT-bot	97%
Proposed	2023	Hybrid CNN-LSTM	CTU-13 IoT-23	98.74% 98.29%

## Data Availability

Some implementations of this research are available online and can be downloaded from https://github.com/adjenna/CNN–LSTM (accessed on 8 June 2023).

## Abbreviations

The following abbreviations are used in this manuscript:

CA	Cybersecurity Analytics
CTI	Cyber Threat Intelligence
DF	Digital Forensics
GDP	Gross Domestic Product
OSINT	Open-Source INTelligence
AI	Artificial Intelligence
DL	Deep Learning
LSTM	Long Short-Term Memory
CNN	Convolutional Neural Network
ML	Machine Learning
FNN	Feedforward Neural Network
WE	Word Embedding
DRNN	Deep Recurrent Neural Network
SVM	Support Vector Machine
BLSTM	Bidirectional Long Short-Term Memory
BiGRU	Bidirectional Gated Recurrent Unit
TP	True Positive
TN	True Negative
FP	False Positive
FN	False Negative
IRC	Internet Relay Chat
P2P	Peer-to-Peer
HTTP	HyperText Transfer Protocol
PCAP	Packet Capture
CSV	Comma-Separated Values)
IoT	Internet of Things
C&C	Command and Control
DDoS	Distributed Denial of Service
List of mathematical symbols	
X	Input data
Y	Output
W	Weight matrix
b	Bias vector
*	Convolution operation
‖	Concatenation operation
σ	Sigmoid activation function
tanh	Hyperbolic tangent activation function
⊗	Cross-correlation operation
⊙	Element-wise multiplication
⊕	Element-wise addition operation
∇	Gradient symbol
*∂*	Partial derivative symbol
θ	Model parameters
α	Mixing coefficient for combining original and synthetic samples in SMOTE
